# Predictors of Spontaneous Rupture of Hepatocellular Carcinoma and Clinical Outcomes Following Hepatectomy

**DOI:** 10.3389/fonc.2022.820867

**Published:** 2022-01-27

**Authors:** Yiran Chen, Deliang Guo, Xinyi Li, Chang Xu, Qian Zhu

**Affiliations:** ^1^ Department of Hepatobiliary and Pancreatic Surgery, Hubei Provincial Clinical Medicine Research Center for Minimally Invasive Diagnosis and Treatment of Hepatobiliary and Pancreatic Diseases, Zhongnan Hospital of Wuhan University, Wuhan, China; ^2^ Department of Anesthesiology, Zhongnan Hospital of Wuhan University, Wuhan, China; ^3^ Second Department of Biliary Surgery, Eastern Hepatobiliary Surgery Hospital of Naval Medical University, Shanghai, China

**Keywords:** tumor of the liver, rupture of hepatocellular carcinoma, mechanism of rupture, treatment, prognosis

## Abstract

**Objective:**

To explore the independent predictive factors of spontaneous tumor rupture (STR) in patients undergoing curative resection of hepatocellular carcinoma (HCC), and to evaluate the impact of STRHCC on long-term survival after hepatectomy.

**Methods:**

The clinicopathological parameters of 106 patients with STRHCC and 201 patients with non-ruptured HCC who underwent hepatectomy from January 2007 to November 2011 at the Eastern Hepatobiliary Surgery Hospital and Zhongnan Hospital of Wuhan University were analyzed using propensity score matching (PSM) and a logistic regression model.

**Results:**

Factors including hypertension, cirrhosis, total bilirubin (TB), tumor size, and ascites were independent predictors of STR. For all 307 HCC patients, the 1-, 3- and 5-year overall survival (OS) rates were 54.0%, 37.3% and 33.8%, respectively. After PSM, the 1-, 3-, and 5-year OS rates in the ruptured group remained significantly lower at 41.5%, 23.5%, and 17.5% when compared with the non-ruptured group at 70.8%, 47.1%, and 37.6%, respectively, while the 1-, 3-, and 5-year disease-free survival (DFS) rates between the groups did not differ significantly (50.4%, 35.1%, 27.1% vs 55.4%, 38.2%, 27.4%). STRHCC was significantly associated with increased risk of OS, but not of shorter DFS. No significant difference in postoperative morbidity or hospital death was observed between the groups.

**Conclusion:**

Factors including hypertension, liver cirrhosis, higher TB levels, tumor size > 5cm, and ascites are significant predictors of STR. The recurrence rate of patients in the ruptured group was significantly higher than that of patients in the non-ruptured group. STR results in poorer OS but not DFS in patients undergoing curative resection for HCC. STRHCC has no impact on postoperative morbidity and mortality after hepatectomy.

## 1 Introduction

Hepatocellular carcinoma (HCC) is the fourth most commonly occurring cancer and the third commonest cause of cancer-related deaths in China (1, 2). Spontaneous tumor rupture of HCC (STRHCC) is a rare fatal complication with an incidence of 10% to 15% (3–5). Immediate intervention for hemostasis is the main treatment for STRHCC. Assessment should be carried out immediately when the bleeding has been arrested, which includes the overall condition of the patient, liver function, tumor stage, and resectability of the tumor (including the tumor location). Given that STRHCC is a contraindication to liver transplantation, hepatectomy remains the only potential curative intervention. The long-term survival rate of patients undergoing hepatectomy is superior to those undergoing other non-surgical treatments such as local ablative therapy, transhepatic artery embolization or chemoembolization (4). Emergency or staged hepatectomy (embolization or other conservative procedures to achieve haemostasis, followed by surgery) is an effective treatment for STRHCC, and long-term survival can be achieved (2–5). Our study aimed to analyze the risk factors for STR and the survival outcomes of patients undergoing curative resection following STR of HCC.

## 2 Materials and Methods

### 2.1 Patients

**Figure 1:**
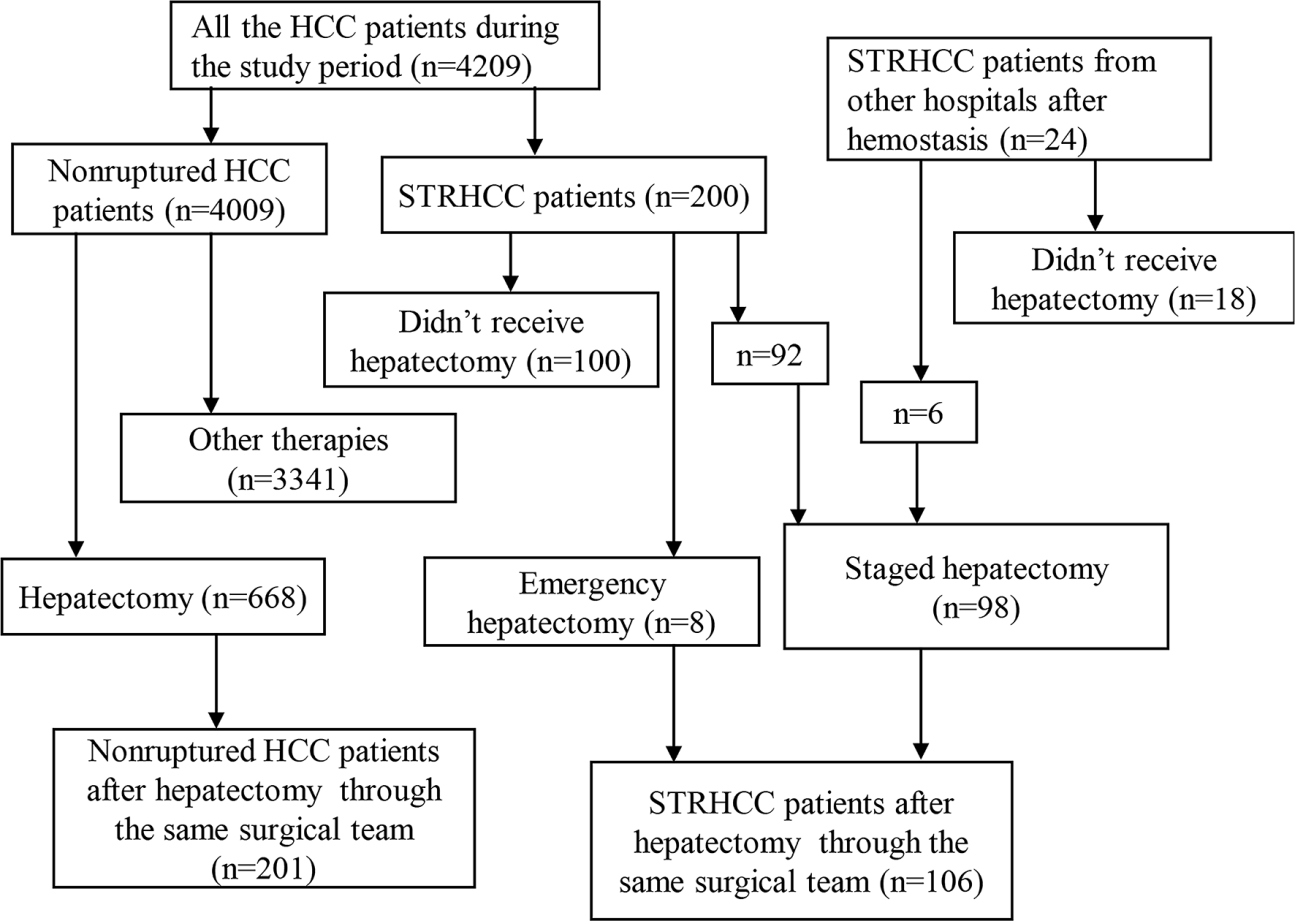
The flow chart illustrated the patient selection process.

This was a retrospective study that included HCC patients that underwent hepatectomy in the Eastern Hepatobiliary Surgery Hospital of Naval Military Medical University and the Zhongnan Hospital of Wuhan University from April 2007 to November 2011 (**Figure 1**). Eligible patients were confirmed to have HCC and liver cirrhosis by histopathological results, or STRHCC by clinical manifestations, physical signs and other diagnostic methods (including Computed Tomography [CT], diagnostic abdominocentesis, ultrasound or angiography), and underwent hepatectomy by the same surgical team. Among the key exclusion criteria were patients with incomplete clinical data, serious complications such as severe cardiovascular disease, preoperative portal vein thrombosis, portosystemic shunting before or during hepatectomy and those undergoing palliative liver resection (undertaken only in patients in whom the tumour was multiple and unresectable, liver functional reserves were inadequate for major hepatectomy). Written informed consents were obtained from all patients. There were 307 patients enrolled on the study. This study was conducted in accordance with the Declaration of Helsinki and approved by the Clinical Research Ethics Committee of the Eastern Hepatobiliary Surgery Hospital of Shanghai and Zhongnan Hospital of Wuhan University in China.

### 2.2 Treatment

For patients with a normal liver function and TNM staging that showed a locally confined liver tumor without metastasis, the liver tumor was assessed for curative resectability. For resectable STRHCC, emergency hepatectomy (the main hemostatic method for STRHCC) was feasible when the liver function and the general condition of the patient met the surgical requirements. For patients not suitable for emergency hepatectomy, staged hepatectomy (staged early hepatectomy: rupture time ≤ 8 d; staged late hepatectomy: rupture time > 8 d) was carried out after other hemostatic interventions (that included liver packing, hepatic artery ligation, suture of the liver parenchyma, local ablative therapy, and alcohol injection). For patients with unstable hemodynamics, the main treatments included close monitoring of vital signs, active anti-shock, transcatheter arterial chemoembolization or transcatheter arterial embolization (TACE/TAE) and supportive treatment. TACE/TAE or emergency exploratory laparotomy was carried out for those with recovered coagulation profiles and when conservative treatments were ineffective.

Emergency hepatectomy: The Pringle method was used to occlude the porta hepatis. Upon evacuation of blood clots from the abdominal cavity, the tumor was evaluated for resectability. After the operation, the abdominal cavity was rinsed with conventional hot distilled water (DWPL) and 5-fluorouracil (5-FU) particles were placed in the omentum.

Staged hepatectomy: Before surgery, CT and magnetic resonance imaging (MRI) were used to assess the location and extent of the tumor for resectability. The Pringle method was used to occlude the porta hepatis for 15 min and released for 5 min. The liver parenchyma was dissected by the classic “clamp method”, and hemostasis was achieved by suturing of the wound and using an argon knife. Anatomical hepatectomy (AH) or non-anatomical hepatectomy (NAH) were selectively performed, with the latter mainly applied to tumors located on the surface of the liver or the junction of multiple hepatic segments, or the liver with severe cirrhosis. Large-scale hepatectomy was defined as resection of three or more Couinaud hepatic segments.

### 2.3 Assessment

#### 2 3.1 Postoperative Complications

Postoperative complications were assessed from the first day after surgery to the day of hospital discharge, including liver failure (TB level >60 μmol/L, prothrombin time >18s or hepatic encephalopathy), cardiopulmonary failure, renal failure, biliary complications, postoperative infections, ascites and pleural effusion requiring drainage.

#### 2.3.2 Follow-Up

Two clinicians who were blinded to this study carried out the follow-ups *via* the combination of postoperative outpatient setting and telephone calls every 4 weeks postoperatively and then every 2 months and 6 months postoperatively, until the 30^th^ of November 2016. Assessments including alpha-fetoprotein (AFP), CT or MRI, chest radiographs, and positron emission tomography (PET-CT) or bone scans (ECT) were performed when indicated. Hospital death was defined as death during hospitalization or within 60 days after surgery. Overall survival (OS) was defined as from the date of surgery to death or the end of follow-up. Disease-free survival (DFS) was defined as from the date of surgery to the time of tumor recurrence or death. The diagnostic criteria for recurrence of HCC were similar to that of the first diagnosis of HCC: patients with hepatitis B (HBV) or hepatitis C (HCV), or with cirrhosis of any cause, underwent ultrasound and serum AFP examination at least every 6 months. Intrahepatic nodules were identified as lesions that were significantly strengthened during the arterial phase and the enhancement was lower than the normal liver parenchyma during the portal venous phase or equilibrium phase (typical imaging characteristic of HCC) on dynamic enhanced MRI, dynamic enhanced CT, ultrasound imaging or liver cell specificity of Gd - EOB - DTPA enhancement MRI contrast agent. HCC was clinically diagnosed when two of the above imagings showed typical imaging characteristics of HCC with the diameter of intrahepatic nodules of ≤ 2cm, or when one conformed to a typical imaging characteristic with the diameter of > 2cm. The management of recurrent HCC was according to the specific situation of recurrence, the reserved function of the liver, and the patient’s general condition. Multidisciplinary treatments including recurrence resection, radiofrequency ablation, transcatheter arterial chemoembolization (TACE), radiotherapy, chemotherapy, or oral sorafenib were carried out for recurrent disease.

### 2.4 Statistical Analysis

Measurement data or count data of patients’ baseline characteristics were expressed as mean ± SD or number of cases (percentage). Respectively, the independent sample t-test or Pearson χ2 test was used for comparisons between the groups. Multivariate adjusted logistic regression analysis was used to explore the risk factors for STRHCC. Direct use of the Kaplan-Meier survival curve discounted the influence of confounders, which might result in errors of survival rate between the groups. However, PSM accurately evaluated the difference in survival rate between the two groups after controlling for the confounders that affected the prognosis except for STRHCC. Therefore, Kaplan-Meier analysis was used after PSM, and a log-rank test was used to compare the survival rate between the groups. In addition, multivariate-adjusted Cox regression analysis and Cox regression analysis after PSM were used and mutually authenticated to determine the effect of STRHCC after hepatectomy on patients’ long-term prognosis. The process of PSM was as follows: (1) patients were divided into the ruptured and the non-ruptured group, (2) rupture or non-rupture was set as the dependent variable (Y) and the other known clinical features as the independent variable (X) to build a logistic regression equation, (3) the rupture probability of each patient was calculated according to the equation, (4) the nearest neighbor matching was used with calipers as the default value to match rupture probability with *P* > 0.05 indicating data balance. Statistical analysis was conducted using the IBM SPSS Statistics 19 software (Statistical Package for the Social Sciences, Inc. Chicago, Illinois) with *P <*0.05 indicating statistical significance.

## 3 Result

There were 4209 patients (3661 males and 548 females) diagnosed with HCC during our study period with the mean age of 49.4 ± 8.7 years old. Of these, 200 (4.8%) had STRHCC. Among 774 patients who underwent liver resection, 106 (13.7%) patients had STRHCC before surgery. A total of 100 patients with ruptured HCC did not proceed with hepatectomy due to several reasons: the tumors were considered unresectable during the preoperative evaluation (51 cases) or during the operation (4 cases); serious derangement of liver functions that were not suitable for surgery (17 cases); the general condition of the patients was poor (10 cases); rejection of surgery (3 cases); and other reasons (15 cases). For our analyses, patients were divided into the ruptured group (n=106) and the non-ruptured group (n=201).

### 3.1 Clinical Characteristics of Patients With STRHCC

The clinical characteristics of the ruptured and the non-ruptured group that underwent hepatectomy were compared (**Table 1**). In the ruptured group, there were 99 males and 7 females with a median age of 46.7 ± 11.3 years old. Between the 2 groups, the age difference was statistically significant (*P*=0.005) but not the gender.

**Table 1:** Comparison of clinical characteristics between HCC patients with STR and those with nonruptured tumors undergoing hepatectomy.

Variate	Before PSM				After PSM			
	Rupture (n=106)	No rupture (n=201)	Statistic^#^	*P* value	Rupture(n=89)	No rupture (n=89)	Statistic^#^	*P* value
Median age (years old)	46.7 ± 11.3	50.6 ± 11.3	-2.832	0.005*	50.1 ± 7.8	50.0 ± 7.5	0.568	0.572
Sex no. (%)			1.541	0.216			0.901	0.342
Male	99 (93.4)	179 (89.1)			81 (91.0)	77 (86.5)		
Female	7 (6.6)	22 (10.9)			8 (9.0)	12 (13.5)		
Hypertension no. (%)	10 (9.4)	6 (3.0)	5.842	0.016*	9 (10.1)	3 (3.4)	4.748	0.073
Cirrhosis no. (%)	89 (84.0)	113 (56.2)	23.737	<0.001*	76 (85.4)	80 (89.9)	0.830	0.362
Child-Pugh grade			11.008	0.001*			0.424	0.515
Grade A	96 (90.6)	199 (99.0)			83 (93.3)	85 (95.5)		
Grade B	10 (9.4)	2 (1)			6 (6.7)	4 (4.5)		
Transfusion no. (%)			19.955	<0.0001*			1.102	0.294
Yes	46 (43.4)	39 (19.4)			47 (52.8)	40 (44.9)		
No	60 (56.6)	162 (80.6)			42 (47.2)	49 (55.1)		
Sudden abdominal pain no. (%)	106 (100.0)	191 (95.0)	3.987	0.046*	89 (100)	84 (94.4)	3.292	0.070
Hb (g/L)	130.3 ± 15.8	132.9 ± 15.2	-1.378	0.169	131.0 ± 15.2	132.3 ± 18.6	-0.684	0.495
TB (μmol/L)	21.8 ± 13.5	16.0 ± 6.7	4.136	<0.001*	22.5 ± 14.2	17.3 ± 6.7	3.106	0.002*
ALB (g/L)	39.7 ± 4.8	40.8 ± 4.2	-2.083	0.039*	39.7 ± 4.9	40.9 ± 4.6	-1.708	0.089
ALT (U/L)	60.3 ± 67.4	59.4 ± 48.3	0.140	0.889	60.7 ± 72.4	57.6 ± 32.8	0.366	0.715
AST (U/L)	67.9 ± 82.6	59.8 ± 47.6	1.089	0.277	67.4 ± 88.6	59.8 ± 38.2	0.750	0.454
PLT (10^9^/L)	160.6 ± 74.1	148.6 ± 64.3	1.413	0.159	159.6 ± 70.3	139.3 ± 57.2	2.111	0.036*
PT (s)	13.0 ± 1.6	13.3 ± 1.5	-1.378	0.169	13.1 ± 1.5	13.3 ± 1.9	-0.684	0.495
HBsAg no. (%)			6.544	0.011*			0.771	0.381
+	98 (92.5)	164 (81.6)			79 (88.8)	75 (84.3)		
–	8 (7.5)	37 (18.4)			10 (11.2)	14 (15.7)		
HBeAg no. (%)			0.752	0.386			0.237	0.627
+	29 (27.4)	46 (22.9)			63 (70.8)	60 (67.4)		
–	77 (72.6)	155 (77.1)			26 (29.2)	29 (32.6)		
AFP>100μg/L no. (%)	75 (70.8)	113 (56.2)	6.178	0.013*	60 (67.4)	49 (55.1)	2.864	0.091
AFP>400μg/L no. (%)	68 (64.2)	93 (46.3)	8.898	0.003*	57 (64.0)	53 (59.6)	0.381	0.537
Edmondson-Steiner grade no. (%)			1.092	0.296			1.304	0.254
Grade III or IV	55 (51.9)	155 (77.1)			50 (56.2)	58 (65.2)		
Grade I or II	51 (48.1)	46 (22.9)			49 (55.1)	41 (46.1)		
Tumor capsular no. (%)			0.552	0.458			1.161	0.281
No or part	42 (39.6)	71 (67.0)			72 (80.9)	66 (74.2)		
Intact	64 (60.4)	130 (64.7)			17 (19.1)	23 (25.8)		
Tumor diameter (cm)	8.6 ± 3.2	7.1 ± 4.5	3.393	0.001*				
Tumor diameter > 5cm no. (%)	89 (84.0)	116 (57.4)	21.556	<0.001*	75 (84.3)	70 (78.7)	0.930	0.335
Ascites no. (%)	51 (48.1)	16 (8.0)	65.583	<0.001*	41 (46.1)	7 (7.9)	32.976	<0.0001*
Macrovascular invasion no. (%)			0.202	0.653			1.103	0.294
Yes	61 (57.5)	121 (60.2)			50 (56.2)	43 (48.3)		
No	45 (42.5)	80 (39.8)			39 (43.8)	46 (51.7)		
Microvascular invasion no. (%)			46.761	<0.0001*			0.672	0.412
Yes	82 (77.4)	73 (36.3)			65 (73.0)	60 (67.4)		
No	24 (22.6)	128 (63.7)			24 (27.0)	29 (32.6)		
The number of tumors no. (%)			91.913	<0.0001*			1.173	0.279
Multiple	26 (24.5)	162 (80.6)			37 (41.6)	30 (33.7)		
Single	80 (75.5)	39 (19.4)			52 (58.4)	59 (66.3)		
Surgical margins no. (%)			7.047	0.008*			1.087	0.297
≤1 cm	17 (16.0)	60 (29.9)			19 (21.3)	25 (28.1)		
>1cm	89 (84.0)	141 (70.1)			70 (78.7)	64 (71.9)		
Extent of hepatectomy			1.893	0.169			0.989	0.320
Major	44 (41.5)	100 (49.8)			7 (7.9)	11 (12.4)		
Minor	62 (58.5)	101 (50.2)			82 (92.1)	78 (87.6)		
Type of hepatectomy no. (%)			5.218	0.022*			0.483	0.487
Anatomical	56 (52.8)	133 (66.2)			20 (22.5)	24 (30.0)		
Non-anatomical	50 (47.2)	68 (33.8)			69 (77.5)	65 (70.0)		
Satellite lesions								
Yes	23 (21.7)	35 (17.4)	0.832	0.362	22 (24.7)	25 (28.1)	0.260	0.610
No	83 (78.3)	166 (82.6)			67 (75.3)	64 (71.9)		
Bleeding volume (mL)	300 (50-5000)	500 (30-8000)	-2.74	0.006*	300 (50-5000)	500 (30-8000)	-0.50	0.615
Operation time (min)	166.8 ± 70.2	165.6 ± 69.0		0.885	171 ± 85	160 ± 73		0.233
T2DM no. (%)			1.709	0.191			0.989	0.320
Yes	10 (9.4)	11 (5.5)			7 (7.9)	11 (12.4)		
No	96 (90.6)	190 (94.5)			82 (92.1)	78 (87.6)		
Hospital death no. (%)	3 (2.8)	3 (1.5)	0.138	0.710	1 (1.1)	1 (1.1)	0.000	1.000
Postoperative complications no. (%)	27 (25.5)	52 (25.9)	0.006	0.939	18 (20.2)	19 (21.3)	3.711	0.054
Clavien grade I	3 (2.8)	5 (2.5)			2 (2.2)	3 (3.4)		
Clavien grade II	13 (12.3)	25 (12.4)			8 (9.0)	9 (10.1)		
Clavien grade III	6 (5.7)	16 (8.0)			3 (3.4)	3 (3.4)		
Clavien grade IV	2 (1.9)	3 (1.5)			3 (3.4)	2 (2.2)		
Clavien grade V	3 (2.8)	3 (1.5)			2 (2.2)	2 (2.2)		
Recurrence no. (%)	95 (89.6)	142 (70.6)	14.197	<0.0001*	78 (87.6)	66 (74.2)	5.235	0.022*

Hb, hemoglobin; TB, total bilirubin; ALB, serum albumin; ALT, alanine aminotransferase; AST：aspartate aminotransferase; PLT, platelet; PT, prothrombin time; HBsAg, hepatitis B virus surface antigen; HBeAg, hepatitis B virus e antigen; AFP, alpha-fetoprotein; T2DM, Type 2 diabetes mellitus.

^#^The statistical values of continuous variables and classified variables were t values of t-test and χ2 values of χ2 test, respectively. *Indicated statistically significant values (*P < 0.05).

The tumor diameter in the ruptured group was significantly larger than that in the non-ruptured group (8.6 ± 3.2 cm vs. 7.1 ± 4.5 cm, t=3.393, *P*=0.001), while other tumor-related clinical features such as tumor capsule, macrovascular invasion, tumor grade, and surgical margin were comparable between the two groups. The intraoperative blood loss volume (300 [50 – 5000) mL vs. 500 [30 – 8000) mL, t=-2.74, *P*=0.006) and the proportion of patients requiring intraoperative blood transfusion (43.4% vs. 19.4%, *P* < 0.0001) of the ruptured group were higher than those of the non-ruptured group. However, no difference in the operation time was observed between the two groups (*P*=0.885). When compared with the non-ruptured group, the proportion of surgical margin ≤1 cm was lower (6.0% vs. 29.9%, χ2 = 7.047, *P*=0.008) while the proportion of NAH was higher in the ruptured group (47.2% vs. 33.8%, χ2 = 5.218, *P*=0.022). A patient in the ruptured group died during hospitalization due to liver failure. After the PSM, there were 89 patients in the ruptured group and 89 patients in the non-ruptured group. There was no significant difference in surgical complications and hospital deaths between the two groups before and after PSM.

### 3.2 Predictors of STRHCC

On univariate analyses, several factors including sudden onset, hypertension, liver cirrhosis, Child-Pugh grade, hemoglobin (Hb), TB, serum albumin (ALB), AFP, hepatitis B virus surface antigen (HBsAg), and tumor size were potential predictors of STRHCC (**Table 1**). Multivariate logistic regression analysis revealed that hypertension, cirrhosis, TB, tumor size and ascites were independent predictors of STRHCC (**Table 2**).

**Table 2:** Multivariate logistic analysis of predictors of STR in HCC patients (before PSM).

Variate	S. E	Wald	HR	95%CI	*P* value
Hypertension (Yes *vs.* No)	0.817	16.451	0.036	0.01-0.18	<0.001
Cirrhosis (Yes *vs.* No)	0.384	18.142	0.195	0.09-0.41	<0.001
Tumor diameter (≤5 cm *vs.* >5 cm)	0.380	19.516	0.187	0.09-0.39	<0.001
TB (≤18.8 umol/L *vs.* >18.8 umol/L)	0.314	9.866	0.373	0.20-0.69	0.002
Ascites (Yes *vs.* No)	0.393	27.499	0.127	0.06-0.28	<0.001

### 3.3 Survival Curves Analysis

During postoperative follow-ups, 92 (86.8%) of 106 STRHCC patients and 124 (61.7%) of 201 non-ruptured HCC patients died. The 1 -, 3 -, and 5-year OS rates were 54.0%, 37.3%, and 33.8%, respectively, with a median survival time of 17 months (95% CI 12.0-25.0). Of the 265 patients who underwent R0 resection, 38(7.5%) developed peritoneal metastasis, including 29 (32.6%, 29/89) in the ruptured group and 9 (5.1%, 9/176) in the non-ruptured group (χ2 = 36.314, *P*<0.001). The 1 -, 3 - and 5-year OS rates of 265 HCC patients who underwent R0 resection were 88.8%, 64.6% and 53.7%, respectively, with a median survival time of 41 months.

There were 178 HCC patients who remained after the PSM, including 89 cases in the ruptured group and 89 cases in the non-ruptured group. The overall follow-up time was 1 to 104 months with a median follow-up time of 35.9 months.

### 3.4 The Effect of STRHCC on Patient Survival

**Figure 2:**
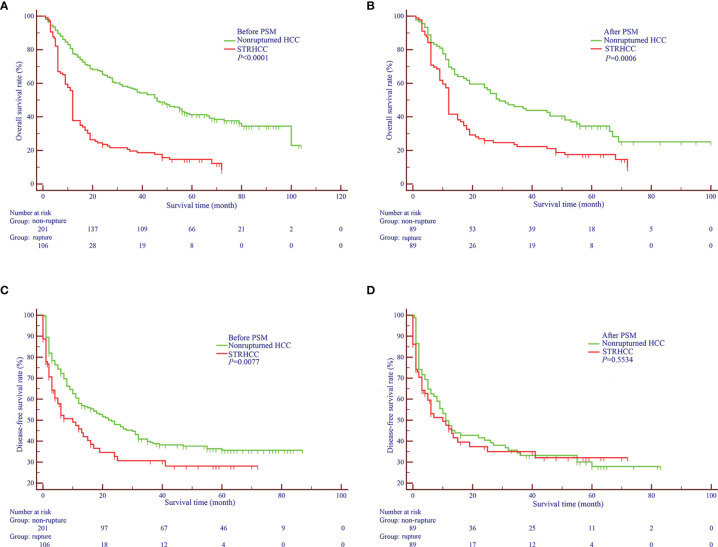
Overall **(A, B)** and Disease-free Survival curves **(C, D)** of nonruptured HCC patients and STRHCC patients before and after PSM.

Before PSM, the 1 -, 3 - and 5-year OS rates of patients in the ruptured group (106 cases) were 37.7%, 19.6%, 14.7%, respectively, and the non-ruptured group (201 cases) were 82.8%, 58.3%, 43.0%, respectively (**Figure 2A**). Furthermore, the 1 -, 3 - and 5-year DFS rates of patients in the ruptured group were 44.5%, 29.7%, 19.4%, respectively, and the non-ruptured group were 66.6%, 44.1%, 30.1%, respectively (**Figure 2B**). Cox regression analysis showed that STRHCC was an independent prognostic factor for OS (HR 0.181, 95% CI 1.324-2.694, *P*<0.001) but not the DFS (HR 0.945, 95% CI 0.635-1.407, *P*=0.782) in 307 HCC patients (**Table 3**). Operative morbidity and mortality rates were comparable after emergency and staged procedures. Although univariable analyses showed that overall survival and DFS after emergency hepatectomy were poorer than after staged hepatectomy (P =0.016 and P =0.025 respectively), the differences were not significant in multivariable analyses.

**Table 3:** Cox regression analysis of prognostic factors of OS and DFS in 307 HCC patients (before PSM).

Variates	SE	Wald	Exp(B)	95%CI	*P* value
OS					
Tumor diameter (≥ 5cm *vs.* < 5cm)	0.183	9.047	1.736	1.212-2.486	0.003
AFP (≥400μg/L *vs.* 400< μg/L)	0.153	9.251	1.591	1.179-2.145	0.002
The number of tumor (multiple *vs.* single)	0.169	9.704	1.692	1.215-2.355	0.002
Microvascular invasion (Yes *vs.* No)	0.208	16.134	2.302	1.533-3.459	<0.001
Child-Pugh grade (A *vs.* B)	0.417	4.068	0.431	0.190-0.977	0.044
STRHCC (Yes *vs.* No)	0.181	12.328	1.889	1.324-2.694	<0.001
DFS					
Tumor diameter (≥5 cm *vs.* <5 *cm*)	0.198	12.022	1.985	1.347-2.926	0.001
AFP (≥400μg/L *vs.* 400<μg/L)	0.167	4.927	1.448	1.044-2.009	0.026
Microvascular invasion (Yes *vs.* No)	0.225	9.622	2.009	1.293-3.121	0.002
STRHCC (Yes *vs.* No)	0.203	0.077	0.945	0.635-1.407	0.782

OS, overall survival; DFS, disease-free survival.

After PSM, the 1 -, 3 - and 5-year OS rates of patients in the ruptured group (89 cases) were 41.5%, 23.5%, 17.5%, respectively, and the non-ruptured group (89 cases) were 70.8%, 47.1%, 37.6%, respectively (**Figure 2C**). Furthermore, the 1 -, 3 - and 5-year DFS rates of patients in the ruptured group were 50.4%, 35.1%, 27.1% respectively, and the non-ruptured group were 55.4%, 38.2% and 27.4%, respectively (**Figure 2D**). Cox regression analysis showed that STRHCC was an independent prognostic factor for OS (HR 1.769, 95% CI 1.524–3.184, *P*=0.001) but not for DFS (*P*>0.05) in 178 HCC patients (**Table 4**).

**Table 4:** Univariate and multivariate regression analysis of prognostic factors for OS and DFS in 178 HCC patients after PSM.

Variate	OS				DFS			
	Univariate		Multivariate		Univariate		Multivariate	
	HR (95% CI)	*P* value	HR (95% CI)	*P* value	HR (95% CI)	*P* value	HR (95% CI)	*P* value
Age	1.013 (0.842-1.083)	0.542			1.010 (0.994-1.024)	0.782		
Sex (male *vs.* female)	1.546 (1.121-2.281)	0.008			1.199 (0.808-1.781)	0.397		
HBsAg	1.101 (0.763-1.379)	0.415			1.001 (0.997-1.006)	0.493		
HBeAg	1.103 (0.784-1.628)	0.401			1.125 (0.815-1.361)	0.381		
TB	0.991 (0.977-1.006)	0.231			1.046 (0.829-1.715)	0.104		
ALB	0.851 (0.624-1.161)	0.309			1.149 (0.902-1.218)	0.351		
ALT	1.014 (0.996-1.006)	0.812			0.989 (0.961-1.019)	0.476		
INR	1.073 (0.824-1.255)	0.103			0.992 (0.825-1.019)	0.529		
PLT	1.015 (0.992-1.018)	0.621			1.339 (0.941-1.905)	0.138		
AFP>400μg/L	1.759 (1.251-2.738)	0.003	1.431 (1.261-1.838)	0.035	1.692 (1.231-3.306)	0.005	1.012 (0.986-1.023)	0.446
Transfusion	1.355 (1.835-2.335)	0.006			1.104 (0.821-1.136)	0.403		
Edmondson-Steiner grade (grade III or IV)	1.049 (0.840-1.310)	0.671			1.515 (1.393-3.052)	0.004	0.946 (0.649-1.381)	0.780
Cirrhosis	1.219 (1.152-3.178)	0.017			1.126 (0.736-1.462)	0.105		
Child-Pugh grade	2.163 (1.680-2.786)	0.001			1.122 (0.804-1.337)	0.402		
Tumor capsular	0.742 (0.656-1.084)	0.183			1.441 (1.274-2.783)	0.037	1.117 (0.831-1.356)	0.236
Tumor diameter	2.262 (1.792-2.855)	<0.001	1.734 (1.274-4.158)	0.002	1.843 (1.235-3.173)	0.007	1.502 (1.361-3.714)	0.004
Macrovascular invasion	2.158 (1.831-3.162)	<0.001			1.425 (1.281-2.821)	0.014	1.967 (1.245-4.681)	<0.001
Microvascular invasion	1.414 (1.106-1.807)	0.006	1.694 (1.310-2.653)	0.025	1.634 (1.221-2.385)	0.023	1.147 (1.012-2.142)	0.126
The number of tumors	1.723 (1.301-2.281)	<0.001			1.227 (1.157-2.418)	0.067		
Type of hepatectomy (AH *vs.* NAH)	1.672 (1.521-2.058)	0.015			1.034 (0.856-2.217)	0.326		
Surgical margins	1.849 (1.621-2.109)	<0.001	1.565 (1.205-2.034)	0.022	1.712 (1.225-3.264)	0.005	1.437 (1.127-3.013)	0.024
T2DM	1.049 (0.840-1.310)	0.671			1.015 (0.956-1.735)	0.426		
STRHCC	1.842 (1.592-3.187)	0.002	1.769 (1.524-3.184)	0.001	1.058 (0.911-1.527)	0.456		

INR, international normalized ratio; AH, anatomical hepatectomy; NAH, non-anatomical hepatectomy.

## 4 Discussion

### 4.1 Epidemiology and Clinical Characteristics of STRHCC

Previous studies have reported that the incidence of STRHCC exhibits regional differences with 10%-15% in HCC patients (1, 4). However, our study showed that STRHCC was more common in HCC patients with poorly preserved hepatic function (worse than Child-Pugh grade B), and especially young HCC patients with HBV infection and cirrhosis. These findings suggest heterogeneity of the patient population with STRHCC, and that the clinicopathological parameters associated with STRHCC also differ among the subgroups.

HCC patients that present with sudden onset of upper abdominal pain coupled with unstable hemodynamics as a result of circulatory shock are the most common clinical manifestations and can almost always be diagnosed with STRHCC. In our study, patients in the ruptured group had more typical clinical symptoms and a significantly higher proportion with liver cirrhosis compared with patients in the non-ruptured group. The recurrence rate of patients in the ruptured group was significantly higher than that of patients in the non-ruptured group. Also, STRHCC was associated with hypertension, ascites, higher Child-Pugh grade, lower ALB level, HBV infection and larger tumor, which were consistent with other studies (6, 7). Contrary to the study by Yeh et al. (6), sudden abdominal pain (univariate analysis, *P*=0.046) was not an independent predictor of STRHCC patients in our cohort. This might be attributed to patient selection in our study that all the included STRHCC patients were operated on by the single liver surgical team.

### 4.2 Risk Factors and Surgical Treatment of STRHCC

From our analyses, hypertension and liver cirrhosis were identified as independent risk factors for STRHCC. Hypertension may directly increase the pressure in the tumor, leading to rupture of blood vessels in tumors with consequent uncontrollable bleeding. When bleeding is by abnormal coagulative function due to cirrhosis, hemostasis will be more difficult to achieve, which potentiates the progression to STRHCC (8).

The management of STRHCC includes hemostasis and hepatectomy. The resectability of a HCC that has ruptured is determined by the location of the tumor in relation to intrahepatic large vessels, residual liver volume, and patient factors including cirrhosis and portal hypertension. Certainly, hepatectomy for STRHCC patients with cirrhosis is technically challenging. It has been reported that ~60.7% to 97.3% of STRHCC patients are with cirrhosis, and only ~12.5% to 30.6% of patients could receive hepatectomy (4). In our cohort, 89 (84.0%) of 106 STRHCC patients with cirrhosis underwent hepatectomy. In recent years, improvements in techniques of liver surgery have made hepatectomy feasible which provides the only chance for a cure for patients with STRHCC.

### 4.3 Clinical Prognosis of STRHCC Patients

In patients with STRHCC, favorable long-term survival following emergency hepatectomy has long been established (9). Our study demonstrated that patients undergoing hepatectomy for STRHCC had a longer OS and DFS, although the OS appeared worse than that of non-ruptured HCC patients. Although numerous studies have reported that STRHCC is an independent but poor prognostic factor after hepatectomy, it remains controversial and there is a need for this to be validated by a prospective study or large-sample clinical cohort study (5, 6). The study by Yeh et al. (6) indicated that the DFS of STRHCC patients was worse than that of non-ruptured HCC patients, but no significant difference in the OS was observed between the two groups. On the contrary, our findings revealed that STRHCC reduced the OS of HCC patients and predicted poor prognosis but not the DFS after hepatectomy. These differences in the findings could be attributed to the surgeons in the two centers of our study having richer experience in liver resection, whereby the proportion of liver resection in our study was significantly higher than most other liver surgical centers (5, 9). This is further reflected in the superior 1-, 3-, and 5-year OS and DFS rates of STRHCC patients after hepatectomy in our cohort than that of the reported prognosis (5, 6).

In our study, no significant differences were observed in the incidence of postoperative complications and perioperative mortality between STRHCC and non-ruptured HCC patients, suggesting no increased adverse events of hepatectomy for STRHCC when performed by experienced surgeons. Intraoperative tumor spread is not uncommon in hepatectomy (2). To prevent abdominal tumor implantation and metastasis, we routinely rinsed the enterocoelia with DWPL and placed 5-FU tablets. DWPL removes cancer cells and thus, delaying tumor recurrence and leading to a better survival prognosis of STRHCC patients (10, 11). A randomized controlled trial (RCT) has confirmed the benefits of 5-FU as postoperative adjuvant therapy, which significantly extends the OS and DFS in patients with advanced HCC (12). Also, the proportion of surgical margin ≤ 1cm in the ruptured group was significantly lower than that in the non-ruptured group. Furthermore, intraoperative evaluation of STRHCC revealed much larger tumors than preoperative evaluations and intraperitoneal implantation metastasis was commonly found. After R0 resection, the risk of peritoneal implantation metastasis was significantly higher in the ruptured group than in the non-ruptured group.

Studies have demonstrated that HCC invasion of the hepatic vein or its branches may obstruct the outflow tract of tumor blood vessels, resulting in hepatic congestion as blood continues to flow into the tumor through the hepatic artery, leading to increased pressure in the tumor and consequently a rupture (4). Nevertheless, our study revealed no difference in the proportion of macrovascular invasion between the ruptured and non-ruptured groups. Immunosuppression induced by perioperative blood transfusion can shorten the DFS of HCC patients (13).

The study by Battula et al. (14) has shown that multiple tumors and tumor size directly predict the survival prognosis. Also, Kirikoshi et al. (15) have demonstrated that tumor size was an independent prognostic factor for the long-term survival of STRHCC patients after TAE. Consistently, our study revealed that tumor size was an independent prognostic factor for the long-term survival of STRHCC patients after hepatectomy. However, we found that HBsAg (+) was not a prognostic factor for OS and DFS of patients with HBV-associated HCC after hepatectomy, contrary to the studies by Sun et al. and Hung et al. (16–20) The prognostic value of HBsAg has been reported variably (2, 20), which may be attributed to the different HBV-DNA copy coefficients of HBsAg (+) patients.

There were several limitations to our study. Firstly, this was a retrospective study. Therefore, we applied PSM to reduce the selective bias caused by confounders and ensure balanced comparability of baseline data between the groups, which makes our results close to that of a RCT study. Secondly, the HBV infection rate in our cohort was significantly higher than that reported in the European and American countries and Japan. Therefore, external validation of our findings is required *via* an international multi-center collaborative study. Furthermore, the surgeons’ extensive experience of hepatectomy in our study may have contributed significantly to the superior clinical outcomes, which is unlikely to reflect the overall national survival statistics of patients with STRHCC. Finally, According to multivariable analysis, tumor diameter < 5cm, AFP < 400μg/L, single tumor, and absence of micro-/macrovascular invasion, as well as the Child-Pugh grade A, increased the odds of long-term survival following liver resection. Future studies are required to establish an effective prognostic nomogram for STRHCC after hepatectomy, to determine whether this model provides more-accurate prediction of patient survival when compared with the currently available staging systems.

## 5 Conclusion

Factors including hypertension, cirrhosis, high level TB, tumor diameter > 5cm and ascites are independent predictors of STRHCC. The recurrence rate of patients in the ruptured group was significantly higher than that of patients in the non-ruptured group. STRHCC itself is an independent prognostic factor for OS but not for DFS of HCC patients after hepatectomy. Hepatectomy after STRHCC is safe, given no increase in the incidence of perioperative complications and mortality.

## Data Availability Statement

The original contributions presented in the study are included in the article/supplementary material. Further inquiries can be directed to the corresponding author.

## Ethics Statement

The studies involving human participants were reviewed and approved by the Clinical Research Ethics Committee of the Eastern Hepatobiliary Surgery Hospital of Shanghai and Zhongnan Hospital of Wuhan University in China. The patients/participants provided their written informed consent to participate in this study.

## Author Contributions

Conception: QZ. Study design: YC, DG, XL, CX, and QZ. Data collection and acquisition: YC, DG, XL, and CX. Data analysis: QZ. Manuscript preparation: QZ. Critical revision: YC, DG, XL, CX, and QZ. All the authors reviewed the paper and approved the final version.

## Funding

This work was supported by the Program of Excellent Doctoral (Postdoctoral) of Zhongnan Hospital of Wuhan University (Grant No. ZNYB2019007), the National Natural Science Foundation of China (Grant No. 82002589), and the Cancer Research and Transformation Platform Project of Zhongnan Hospital of Wuhan University (Grant No. ZLYNXM202004).

## Conflict of Interest

The authors declare that the research was conducted in the absence of any commercial or financial relationships that could be construed as a potential conflict of interest.

## Publisher’s Note

All claims expressed in this article are solely those of the authors and do not necessarily represent those of their affiliated organizations, or those of the publisher, the editors and the reviewers. Any product that may be evaluated in this article, or claim that may be made by its manufacturer, is not guaranteed or endorsed by the publisher.

## Abbreviations

STR, spontaneous tumor rupture; HCC, hepatocellular carcinoma; PSM, propensity score matching; TB, total bilirubin; OS, overall survival; DFS, disease-free survival; CT, computed tomography; TAE, transcatheter arterial embolization; DWPL, distilled water; 5-FU, 5-fluorouracil; MRI, magnetic resonance imaging; AH, anatomical hepatectomy; NAH, non-anatomical hepatectomy; AFP, alpha-fetoprotein; PET-CT, positron emission tomography; ECT, bone scans; HBV, hepatitis B; HCV, hepatitis C; TACE, transcatheter arterial chemoembolization; Hb, hemoglobin; ALB, serum albumin; ALT, alanine aminotransferase; AST; aspartate aminotransferase; PLT, platelet; PT, prothrombin time; HBsAg, hepatitis B virus surface antigen; HBeAg, hepatitis B virus e antigen; T2DM, Type 2 diabetes mellitus; INR, international normalized ratio.
